# Ex vivo recovery and activation of dysfunctional, anergic, monocyte-derived dendritic cells from patients with operable breast cancer: critical role of IFN-alpha

**DOI:** 10.1186/1471-2172-9-32

**Published:** 2008-06-27

**Authors:** Sukchai Satthaporn, Mark M Aloysius, Richard A Robins, Chandan Verma, Suebwong Chuthapisith, Alasdair J Mckechnie, Mohamad El-Sheemy, Wichai Vassanasiri, David Valerio, David Clark, Jibril A Jibril, Oleg Eremin

**Affiliations:** 1 Section of Surgery, Queen's Medical Centre, Nottingham University Hospitals, Nottingham, UK; 2 Department of Immunology, Queen's Medical Centre, Nottingham University Hospitals, Nottingham, UK; 3 Lincoln Breast Unit, Lincoln County Hospital, Lincoln, UK; 4 Research and Development Department, Lincoln County Hospital, Lincoln, UK; 5 Department of Forensic and Biomedical Sciences, Faculty of Health, Life and Social Sciences, University of Lincoln, UK

## Abstract

**Background:**

Dendritic cells (DCs) play a crucial role in initiating effective cell-mediated immune responses, but are dysfunctional and anergic in breast cancer. Reversal of this dysfunction and establishment of optimal DC function is a key prerequisite for the induction of effective anti-cancer immune responses.

**Results:**

Peripheral blood DCs (PBDCs) and lymph node DCs (LNDCs) generated *in vitro *from adherent cultures of peripheral blood monocytes (PBMs) and lymph node monocytes (LNMs), respectively, using the 4 cytokine conditioned medium (CCM) (GM-CSF+IL-4+TNF-α+IFN-α) or 3 CCM (GM-CSF+IL-4+TNF-α) demonstrated a significantly higher degree of recovery and functional capacity in a mixed lymphocyte DC reaction (MLDCR, p < 0.001), expressed significantly higher levels of HLA-DR, CD86, compared with 2 CCM (GM-CSF+IL-4) or medium alone generated DCs from PBMs and LNMs (p < 0.001). The PBDCs generated with 3 CCM or 4 CCM showed a significantly (p < 0.001) enhanced macropinocytotic capability (dextran particles) and induced increased production and secretion of interleukin-12p40 (IL-12p40) *in vitro *(p < 0.001), compared with PBDCs generated from monocytes using 2 CCM or medium alone. Lipopolysaccharide (LPS) stimulation of PBDCs generated with 4 CCM demonstrated enhanced secretion of IL-6 but not IL-12p70, compared with control DCs unstimulated with LPS (p < 0.001).

**Conclusion:**

Dysfunctional and anergic PBDCs and LNDCs from patients with operable breast cancer can be optimally reversed by *ex vivo *culturing of precursor adherent monocytes using a 4 CCM containing IFN-α. Maximal immunophenotypic recovery and functional reactivation of DCs is seen in the presence of IFN-α. However, 4 CCM containing IFN-α generated-PBDCs, do not produce and secrete IL-12p70 *in vitro*.

## Background

A defective host anti-tumour immune response is an important mechanism enabling tumours to evade control and lead to progressive tumour growth [1-3]. Induction of an effective anti-tumour immune response requires the active and integrated participation of host antigen presenting cells (APCs) responsible for the presentation of tumour-associated antigens (TAAgs) [1,2]. Dendritic cells (DCs) are the most potent APCs. They are derived from haematopoietic progenitors and play a crucial role in anti-tumour immunity by taking up TAAgs and stimulating Ag-specific T cells [4]. The transition of DCs from Ag-processing to Ag-presenting cells is accompanied by defined alterations in their surface membrane protein expression [4]. Up-regulation of class I and class II major histocompatibility (MHC) proteins, CD80 and CD86 co-stimulatory molecules, and CD40 adhesion molecule expression enhances the ability of DCs to present Ag to naïve T lymphocytes in secondary lymphoid compartments and induce their activation and generation of peptide-specific cytotoxic T lymphocytes (CTLs) [5]. Activated and mature DCs also produce interleukin-12 (IL-12), which stimulates CD4+ T helper 1 (Th1) cell activation and development, which is important for the *in situ *(paracortex of lymph nodes) expansion of induced anti-tumour CTLs [6-8].

Several groups have described defective function of DCs in tumour bearing mice and in patients with different types of solid cancers [1,2,9-15]. The major findings from these studies were the lack of expression of class II MHC proteins, and co-stimulatory molecules on DCs, consistent with an immature phenotype and functionally inactive status. These DCs demonstrate a reduced capability to secrete key lymphocytic immunoregulatory and stimulatory cytokines [9,11,16]. Immature DCs also directly suppress antigen-specific T cell responses, thereby, inducing immune tolerance to TAAgs and T cell anergy[17]. Dendritic cells isolated from tumour bearing mice also demonstrate a decreased expression of CD86 and MHC class II molecules, as well as certain adhesion molecules; in parallel with these changes is a reduced capacity to stimulate allogeneic T lymphocytes in the mixed lymphocyte dendritic cell reaction (MLDCR) assay *in vitro*. These cells lack the capability to induce effective, peptide-specific and anti-tumour cytotoxic immune responses [13,14,17]. We have previously shown in women with operable breast cancer, that both peripheral blood (PBDCs) and tumour-draining axillary lymph node DCs (LNDCs) were dysfunctional and switched off, as assessed by phenotypic profile (HLA-DR, CD86 and CD40) and functional assays *in vitro *(MLDCR, autologous purified protein derivative (PPD) stimulation assay) [15]. The data presented in this article shows that it is possible to reverse this defective DC function by generating DCs by *in vitro *cultures of peripheral blood monocytes (PBMs) and lymph node monocytes (LNMs) from these patients, with specific combinations of cytokines. Reversal of DC dysfunction (blood and lymph node) and the establishment of optimal DC activity is a key element in any strategy employed to generate an effective and clinically relevant anti-cancer immune response. This immunotherapeutic approach requires the generation *in vitro *of large quantities of functionally and phenotypically active DCs, or, more practically in humans, through recovery of dysfunctional DCs *in vitro *and their subsequent pulsing with peptides/TAAgs and used in immunotherapeutic vaccination [18]. A number of autologous DC vaccination approaches have been used recently but with limited success [19].

The generation of human DCs from plastic-adherent peripheral blood monocytic cultures has traditionally relied on two crucial cytokines, granulocyte-monocyte colony stimulating factor (GM-CSF) and interleukin-4 (IL-4) [20-22]. However, this regimen generates mostly immature DCs, with a CD40 and CD86^low/int^, CD1a^high ^phenotype after one week of *in vitro *culture, even when tumour necrosis factor-α (TNF-α) is included in the culture medium [20-22]. Thus, additional cytokines are needed to facilitate the further activation and maturation of the cells into CD1a^low^, CD40^high^, CD 80^high^, CD86^high ^and HLA-DR^high^cells. Cytokines required for DC terminal differentiation from healthy volunteers have been described previously, which includes interferon-α (IFN-α) but there is very little published on its role in DC generation from monocytes of operable breast cancer patients [23-25]. Macrophage-conditioned medium has been shown to be the most potent inducer of the human DC maturation from immature precursors; however, no single cytokine has emerged to be indispensable in this process, as assessed by using cytokine-neutralizing antibodies [26-28].

Interferon-α is a potent immunoregulatory cytokine. It is secreted early during an immune response by monocytes/macrophages and other less-defined monocytes of myeloid origin resembling immature DCs [29-32]. It inhibits viral replication and aids in stimulating T cell-mediated immune responses against foreign pathogens[29]. Interferon-α has also been used clinically for the treatment of chronic viral infections such as hepatitis and in patients with solid and haematopoietic tumours [33,34]. Type I IFN (IFN-α2a) may enhance the terminal differentiation of CD1a^+ ^immature DCs, derived from human blood CD34^+ ^progenitor cells [35].

In the study reported here, we investigated the effect of low levels of IFN-α (comparable to the levels attainable in the serum of patients receiving the cytokine therapeutically), used concurrently with other cytokines, on the activity and maturation of DCs.

## Results

### The immunophenotype of DCs grown in different cytokine conditioned media (CCM)

The starting population of mononuclear cells from blood and lymph nodes were heterogeneous, consisting of CD14^+ ^cells (24 ± 8%), CD3^+ ^cells (63 ± 9%), CD20^+ ^cells (9 ± 4%), and CD56^+ ^cells (12 ± 4%).

**Figure 1:**
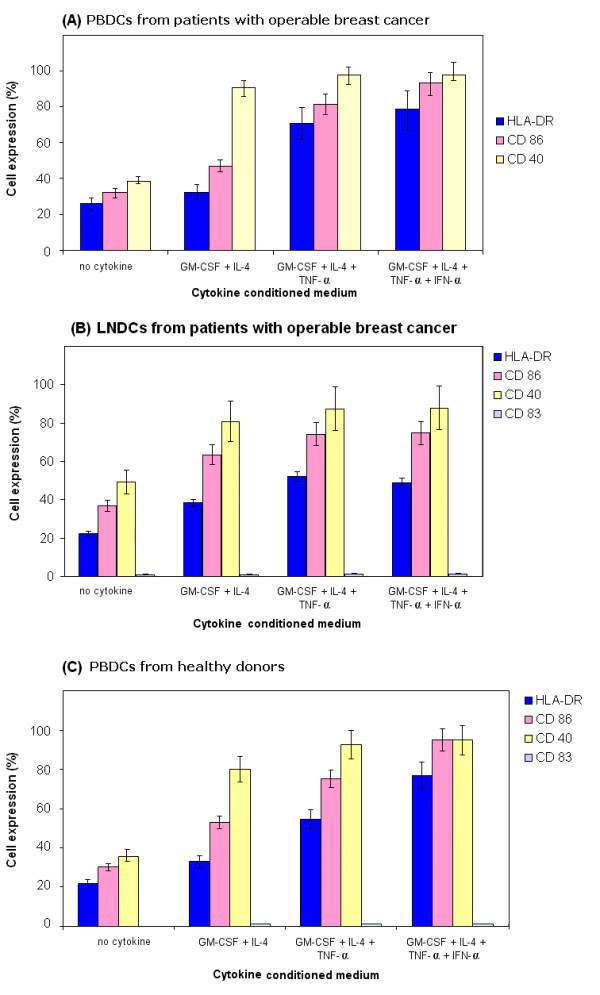
**(A) Percentage of PBDCs (from patients with operable breast cancer) expressing HLA-DR, CD 86 and CD 40**: Statistically significant increase in expression of HLA-DR was observed in the 3 and 4 CCM (cytokine conditioned medium) compared with 2 CCM generated DCs and medium only generated DCs (p < 0.001, ANOVA). Similarly, significant increase in expression of CD86 was observed in the 3 or 4 CCM compared with 2 CCM (cytokine conditioned medium) generated DCs or medium only generated DCs (p < 0.001, ANOVA). However, CD40 expression was increased (p < 0.01, ANOVA) in the 3 or 4 CCM generated DCs compared with medium only DCs. **(B) Percentage of LNDCs (from patients with operable breast cancer) expressing HLA-DR, CD 86 and CD 40: **Statistically significant increase in expression of HLA-DR, CD 86 and CD40 on LNDCs cultured with 3 and 4 CCM compared with medium only generated DCs from patients with breast cancer (p < 0.01, ANOVA). **(C) Percentage of PBDCs (healthy donor) expressing HLA-DR, CD 86 and CD 40: **Significant increase in expression of HLA-DR was observed in the 3 and 4 CCM (cytokine conditioned medium) compared with 2 CCM generated DCs and medium only generated DCs (p < 0.001, ANOVA). However, significant (p < 0.01, ANOVA) increase in expression of CD86 was observed in the 3 or 4 CCM (cytokine conditioned medium) compared with medium only generated DCs. Similarly, CD40 expression was significantly increased in 3 or 4 CCM generated DCs compared with medium only generated DCs. Results shown in 1 A, B and C are represented as mean ± standard deviation (error bars) are from ten different patients or healthy donors. Lineage-negative cells were selectively gated and analyzed in all conditions.

Peripheral blood monocytes and LNMs grown in various CCM; 3 CCM (GM-CSF+IL-4+TNF-α) or 4 CCM (GM-CSF+IL-4+TNF-α+ IFN-α), expressed high levels of HLA class II proteins, CD86, consistent with their differentiation into activated DCs compared with DCs cultured with 2 CCM (GM-CSF + IL-4) or medium alone (Figure 1A, B and 1C). The expression of these immunoregulatory proteins was highest on PBDCs (Figure 1A) and LNDCs (Figure 1B) cultured in the presence of 4 CCM compared with 2 CCM and medium only cultured DCs (p < 0.001). The immunophenotypic profile of PBDCs generated from monocyte cultures of healthy donors demonstrated a pattern, comparable to the optimal immunophenotypic profile obtained with 4 CCM (p < 0.001) (Figure 1C). CD83 expression (marker of terminally mature DCs) was expressed by a small number of DCs in each CCM culture, even with DC generated in the presence of 4 CCM.

### IFN-α increases the CD86 expression on immature DCs

**Figure 2:**
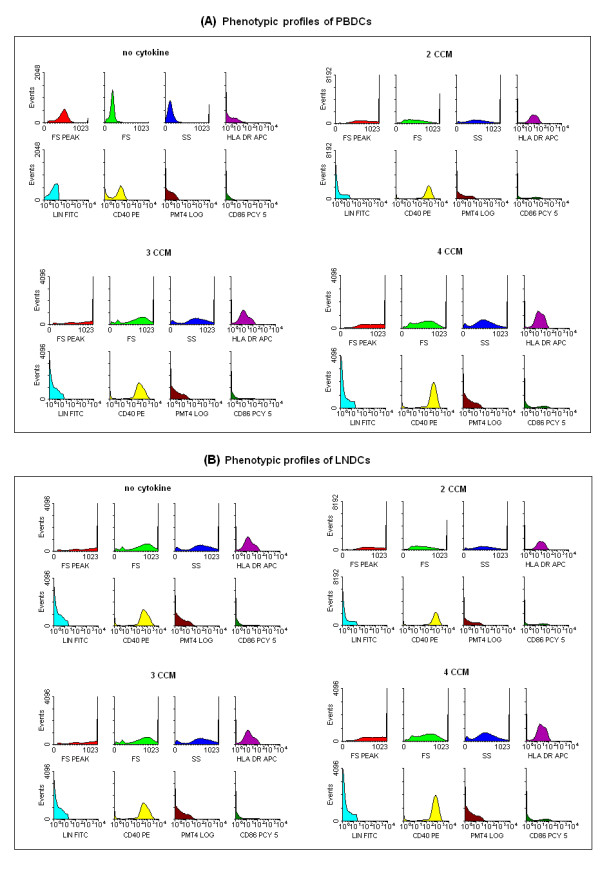
**Phenotypic profile of PBDCS (A) and LNDCs (B) generated with medium alone (no cytokine), 2, 3 and 4 CCM**: Representative maximal fluorescent intensity from flow cytometry of the increasing expression of HLA-DR, CD 40 and CD 86 in different *in vitro *conditions. (Lineage negative cells were gated and analyzed only)

We were interested in whether low levels of IFN-α (500 IU/ml), added at the initiation of DC development along with GM-CSF, IL-4, and TNF-α, had any effect on the DC maturation process. During a 5–7 day culture period of adherent monocytes, maturing DCs become non-adherent and appeared as aggregates of floating cells. The majority of cells expressed high levels of HLA class II as well as CD86, a well-documented marker for activated DCs [36]. In the experiment shown in Figure 1A,B and 1C, monocytes were cultured in the presence or absence of IFN-α (500 IU/ml) for 7 days and were then stained for HLA-DR and CD86 expression. Cells exhibiting a high forward scatter and increased side scatter were gated. This subset contained all the class II, and CD86 co-expressing cells found in the cultures. The increase in the CD86 expression during DC induction from monocytes was reproducible, both from the blood and regional tumour draining lymph nodes of patients with operable breast cancer. Flowcytometric histograms representative of phenotypic profiles of DCs generated using different CCM are illustrated in Figure 2A and 2B. The inclusion of IFN-α (500 IU/ml) at the beginning of the culture led to a 2–3 fold increase in the total number of activated CD86^high ^DCs.

### DCs generated with IFN-α are capable of enhanced dextran uptake via mannose receptors

**Figure 3:**
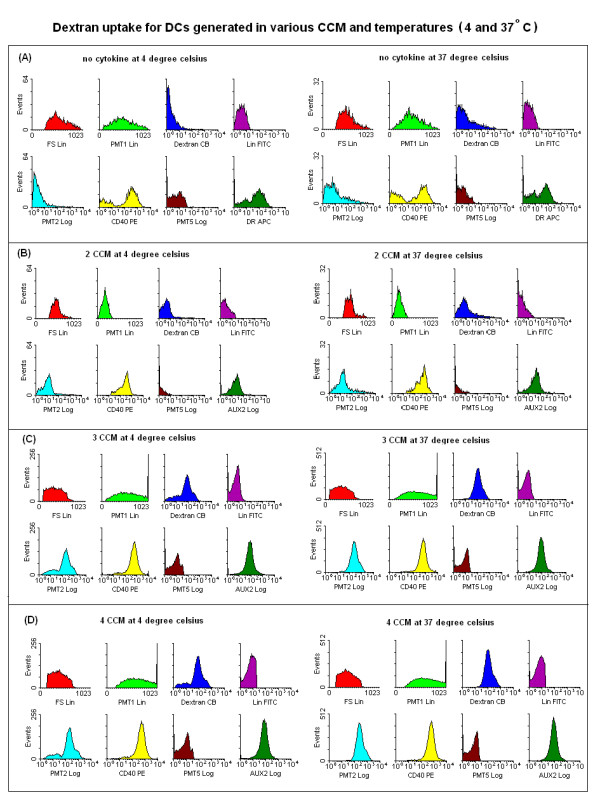
**Phagocytosis (dextran uptake) for DCs generated in the presence of medium only (A), 2 CCM (B), 3 CCM (C) and 4 CCM (D)**: Representative maximal fluorescent intensity of mannose receptors in putative Ag uptake by incubation with Cascade Blue-conjugated dextran in different *in vitro *conditions (at 4°C and 37°C). Lineage negative cells were gated and analyzed only. 0

**Figure 4:**
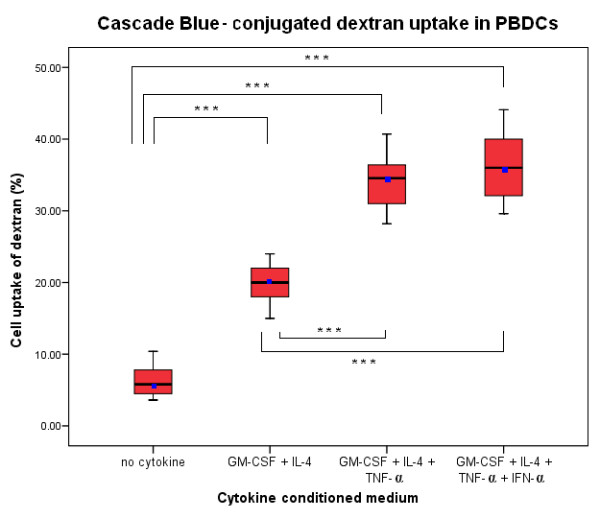
**Box plot showing mannose receptors in putative antigen uptake by incubation with Cascade Blue – conjugated dextran in different *in vitro *conditions of PBDCs generated from 10 different patients**: Percentage of uptake by cells of Cascade Blue conjugated-dextran were calculated from Lin-cells (Lin-cells were gated and analyzed). PBDCs grown in 4 CCM (GM-CSF+IL-4+TNF-α+IFN-α) or 3CCM (GM-CSF+IL-4+TNF-α) compared with medium only generated PBDCs or 2 CCM (GM-CSF+IL-4), both demonstrated significantly higher levels of dextran uptake (*** p < 0.001, ANOVA). Horizontal bars represent mean value for each category. Data shown are median (line), mean (dot), interquartile range (box) and maximum and minimum values (whiskers).

DCs from peripheral blood of 10 breast cancer patients were evaluated for their ability to employ mannose receptors in putative Ag uptake by incubating them with Cascade Blue-conjugated dextran and analysing them by flow cytometry. The true fluorescence produced by dextran uptake was background fluorescence at 4°C subtracted from the fluorescence at 37°C. Flowcytometric histograms representative of this are illustrated in Figure 3A, B, C and 3D. DCs cultured using 3 CCM or 4 CCM, demonstrated significantly higher levels of dextran uptake than those DCs prepared using 2 CCM or with medium alone (p < 0.001) (Figure 4).

### Cytokine production

**Figure 5:**
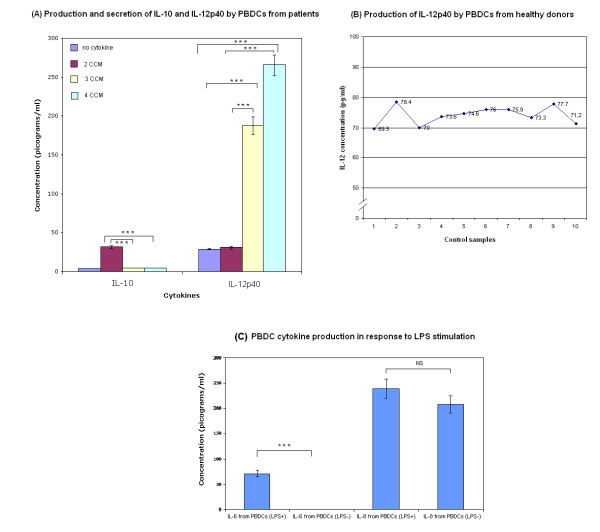
**(A): Differential production of IL-10p40 and IL-12**: Activation/maturation of PBDCs by the 3 and 4 CCM (cytokine conditioned media) leads to significantly increased production and secretion of the pro-inflammatory cytokine IL-12 p40, compared with medium only (*** p < 0.001, ANOVA) and 2 CCM generated DCs (***p < 0.001, ANOVA). In contrast, 3 and 4 CCM generated DCs produced and secreted significantly less inhibitory cytokine IL-10 compared with 2 CCM generated PBDCs (***p < 0.001, ANOVA). Cell free supernatants were obtained on day 7 of culture. Cytokine concentrations were determined by ELISA. The results shown are from ten different patients and are represented as mean ± standard deviation. Error bars represent standard deviations. **(B): Production of IL-12p40 by PBDCs from healthy donors (controls): **The amounts of cytokines in the samples were measured using ELISA. The levels of IL-10 and IL-12 p40 from PBDCs generated from monocytes cultured using all 4 cytokines (4 CCM) from 10 normal healthy donors were evaluated. The mean level of IL-12 p40 was 72.95 ± 4.47, which was significantly (p < 0.01, Student t-test) lower compared with 4 CCM generated PBDCs from breast cancer patients (Mean = 262 ± 15). However, IL-10 was not detectable in the supernatants and hence not shown in this Figure. **(C): Cytokine production by PBDCs generated with 4 CCM from patients with operable breast cancer following stimulation with LPS (1 μg/300μl): **IL-12p70 was undetectable before and after LPS stimulation and hence not shown in this Figure. IL-6 production and secretion was significantly increased after LPS stimulation (*** p < 0.001, Student t-test). NS: Not significant.

The levels of pro-inflammatory or inhibitory cytokines may markedly influence the activation of T lymphocytes. To address this issue, the production of the pro-inflammatory cytokine IL-12 p40 and the inhibitory cytokine IL-10 by PBDCs from patients with operable breast cancer was investigated (Figure 5A). The production and secretion of IL-10 was highest from cultures containing 2 CCM. Using GM-CSF/IL-4/TNF-α ± IFN-α (3 or 4 CCM), however, did not lead to enhanced production and secretion of IL-10. The production of IL-12 p40 was significantly increased in DCs generated with (3 or 4 CCM), when compared with IL-4/GM-CSF alone and control cultures. The highest level was seen in cultures containing IFN-α. We also analysed the levels of IL-10 and IL-12 p40 in culture supernatants of PBDCs generated from normal healthy donors with the optimal 4 CCM (Figure 5B). IL-10, was not produced by PBDCs from healthy donors in contrast to PBDCs from patients with operable breast cancer. Moreover, IL-12 p40 was greater in PBDCs from patients with early breast cancer, generated by IFN-α containing 4 CCM compared with PBDCs generated identically from healthy donors (p < 0.001). IFN-α matured monocyte-derived DCs from healthy donors are known to have impaired production of IL-12 p40 [37].

A further 7 patients with operable breast cancer were recruited and PBDCs were generated with the optimal 4 CCM containing IFN-α, which optimally activates DCs as shown before. The production and secretion of other cytokines (IL-12p70, IL-6 and IL-8) in response to LPS stimulation were studied. IL-12p70 was consistently undetectable after LPS stimulation of PBDCs from all 7 patients. However, IL-6 (but not IL-8) was significantly increased after LPS stimulation (p < 0.001). (Figure 5C)

### The DCs generated with IFN-α are potent activators of an allogeneic MLDCR

**Figure 6:**
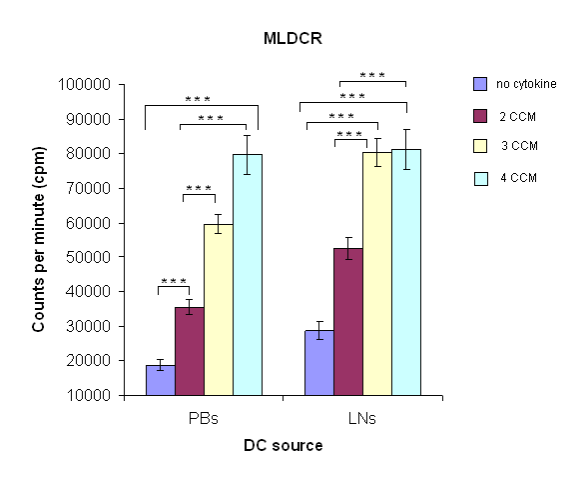
**Increased blastogenesis in the MLDCR**: The stimulatory capacity of DCs.(PB:peripheral blood, LN:lymph node) was estimated from the incorporation of ^3^H-thymidine (shown as cpm). During the last 18 h on the 5^th ^day of *in vitro *culture containing T cells (5 × 10^4 ^cells/well) from a normal volunteer and irradiated DCs (2.5 × 10^3 ^cells/well) from patients with operable breast cancer (PBDCs and LNDCs). DC:T cell ratio was 1:20. LNM and PBM-derived DCs with 3 and 4 CCM milieu showed a significantly increased allogeneic stimulatory capacity compared with 2 CCM (***p < 0.001, ANOVA) and medium only generated DCs (***p < 0.001, ANOVA). Results shown (mean ± standard deviation) are from ten different patients. Error bars represent standard deviations.

The DCs produced by culturing monocytes in a variety of CCM were evaluated for their ability to stimulate T cells in an allogeneic MLDCR. PBDCs from 10 breast cancer patients generated in the presence of 3 and 4 CCMs significantly stimulated allogeneic T lymphocyte proliferation *in vitro *in the MLDCR compared with 2 CCM and medium only generated DCs (p < 0.001). However, the highest level of MLDCR stimulation by PBDCs was seen when all four cytokines (containing IFN-α) were used (Figure 6). In contrast, LNDCs cultured in 4 CCM did not significantly enhance further, the level of stimulation seen with 3 CCM in the MLDCR assays (Figure 6). These results demonstrate that DCs, isolated from the circulation and secondary lymphoid compartments of patients with breast cancer, can be activated *in vitro *with a 4 CCM containing IFN-α, and are highly functional. The activity of these cultured DCs in the allogeneic MLDCR correlates closely with their expression of HLA-DR, CD86, and CD40 immunoregulatory proteins.

## Discussion

A rapidly evolving cancer immunotherapy approach is the induction of TAAg specific, CD8+ CTL-mediated anti-cancer immune response, generated by vaccination with functional and active autologous DCs, pulsed with TAAgs. The host and the tumour *milieu *are hostile to DC maturation and activation, necessitating *ex vivo *manipulation of these cells to resuscitate their impaired function and optimally activate them. After pulsing with TAAg *in vitro*, they can be used in vaccination [17]. Sources of DCs for clinical trials include CD34+ blood or bone marrow-derived precursors, blood DCs and CD14+ monocytes. CD14+monocyte-derived DCs are the most widely used in clinical trials. The 'gold standard' approach, commonly used in many pilot studies, to derive mature DCs from CD14+ cells, is to culture these cells in the presence of IL-4, GM-CSF, IL-1β, TNF-α and PGE2 [38]. However, this approach has been criticized due to low or absent production of IL-12 [39]. Moreover, there is speculation that lack of response may be due to terminally mature DCs (expressing CD83), incapable of inducing efficient CTLs against TAAgs [40]. Alternative DC activation and maturation approaches have been described using IL-13 instead of IL-4, CD40L, poly I:C and FLT3-ligand [38]. The relatively low clinical responses with all these approaches, underpins the current need for novel *ex vivo *DC reactivation techniques, yielding optimally functional and mature DCs.

The aims of our study were two-fold: firstly, to establish that *ex vivo *dysfunctional/switched off DCs from women with early operable breast cancer can be replaced by fully functional DCs generated from monocytes, originating from peripheral blood and the lymph nodes, and secondly, to optimize the *in vitro *conditions to ensure the successful generation of DCs capable of inducing an effective anti-cancer cell-mediated immune response *in vivo *[15].

Our findings demonstrate a very effective *in vitro *approach, to both reversing and substantially activating, the dysfunctional and switched off DCs derived from blood and lymph node monocytes of patients with operable breast cancer. This induction of DC maturation and activation has significant relevance in DC-based immunotherapy for cancer. Previous reports have shown that IFN-α can induce the expression of CD86 and ICAM-1 in murine Mac-1 monocytes/macrophages in a model of diabetes induction *in vivo *[41]. In addition, a number of hepatocellular carcinoma cell lines were shown to respond to IFN-α by inducing CD86 expression [42]. Our results show that the addition of low doses of IFN-α substantially enhanced the expression of CD40^high^, CD86^high^, HLA class II^high ^in DCs generated from adherent cultures of monocytes obtained from blood and lymph nodes of patients with operable breast cancer. Dendritic cells that mature in the presence of IFN-α also exhibit maximal ability to stimulate T cell proliferation in MLDCR and show enhanced macropinocytic function. To the best of our knowledge, this is the first time DCs have been generated and matured from monocytes from patients with operable breast cancer, using a combination of GM-CSF+IL-4+TNF-α and IFN-α.

Our current and previous studies emphasize the necessity of defining the stage of DC activation/maturation when designing strategies and protocols for immunotherapy of cancer, using DC-based immunization regimens. Published data suggests that the capacity to induce a tumour-specific immune response by monocyte-derived DCs *in vivo*, is very much dependent on the degree of DC activation and/or maturation, when used for anti-cancer vaccination [4]. In our study, PBMs and LNMs cultured with IFN-α containing 4 CCM optimally differentiated into activated DCs as demonstrated by immunophenotypic profile and MLDCR,. Enhanced macropinocytosis and IL-12p40 cytokine secretion was seen with the 4 CCM containing IFN-α. The critical role of DCs as APCs is reflected by their high-level of expression of class I and II HLA molecules, adhesion molecules (eg. CD40) and the co-stimulatory molecules CD80 and CD86 [43-48]. Dendritic cells generated with the 4 CCM exhibited the highest percentage of cells with these immunoregulatory proteins. The key adhesion molecules (CD40), expressed on DCs are critical for the superfamily TNFR (tumour necrosis factor receptor) CD40L (CD154) on CD4+T cells. The latter are vital for CD4+T cell licensing and activation through DCs [4,49]. Co-stimulatory molecules (CD80 and CD86) are crucial for optimally enhanced interaction of DCs with CD28 receptor on T lymphocytes and their resultant activation and proliferation. These molecules were also expressed at high (maximal) levels by 3 CCM treated-DCs. The CD83 molecule, a marker for mature terminally differentiated DCs, was expressed by only a minority of the DCs prepared using all four cytokines (4 CCM) [50]. These findings suggest that most of the DCs were not terminally differentiated and, therefore, were likely to be biologically effective *in vivo*. Moreover, cells grown in TNF-α, with or without IFN-α (3 and 4 CCM DCs) had lobulated nuclei and numerous fine cytoplasmic projections consistent with the morphological features of mature and activated DCs. Cells cultured in 2 CCM only, had fewer fine cytoplasmic processes and were smaller with round nuclei, which are features of immature and inactivated DCs.

Different combinations of cytokines using IFN-α have been described in generating DCs from PBMs of healthy volunteers and combinations with this cytokine have been shown to produce DCs of mature phenotype and enhanced function [23,24,51,52].

Our results are at variance with previous studies, which describe DCs derived and matured using IFN-α from PBMs of healthy subjects and patients, when cultured in the presence of TNF-α or other DC-activating agents (e.g. LPS) [53,54]. The immunophenotypic characteristics of the DCs predicted their functional capabilities. For example, PBDCs and LNDCs generated from PBMs and LNMs in different CCM, stimulated allogeneic T lymphocyte proliferation to a variable degree in the MLDCR assay, depending on the cytokine combinations used. DCs concurrently demonstrated higher and differential levels of cells expressing HLA-DR, CD86, and CD40, depending on the CCM used. As in the functional assays, maximal numbers of activated DCs were obtained using the 4 CCM. Moreover, the PBM-generated DCs using 3 or 4 CCM secreted significantly more IL-12 p40 than DCs generated using 2 CCM or just medium alone (no cytokines). In contrast to Svane *et al *we have demonstrated that IL-12p40 secretion was increased in PBM-generated DCs from patients with early malignant breast disease using IFN-α [37]. This may possibly be due to DCs from patients with early (operable) breast cancer being functionally different and responding differentially to cytokine stimulation compared with DCs from healthy volunteers. In advanced breast cancer, TNF-α and polyI:C have also been used before as maturation stimuli and to induce secretion of IL-12 [55].

Interferon-α cultured DCs from adherent PBMs of healthy donors behave differently depending on the timing of exposure to this cytokine. Early exposure before DC differentiation depletes DC precursors, whereas late exposure after differentation confers DC maturity [23,56]. In contrast to this, we have shown that early exposure of PBM-derived DCs from patients with early (operable) breast cancer, to IFN-α, leads to a better phenotype and function. DC maturation with this cytokine combination and effects has not been previously reported in this group of cancer patients, nor has re-activation of LNM-derived DCs, from local tumour draining lymph nodes, been demonstrated.

Dendritic cells have the ability to take up, process and present soluble Ags to autologous T lymphocytes. Antigen uptake by DCs grown in 2 CCM is mediated predominantly by macropinocytosis or binding to mannose receptors [57]. The ability of the activated DCs generated with IFN-α to retain macropinocytosis for antigen uptake is new and contrasts with published evidence that mature DCs lose this ability [57]. Despite a high percentage of our DCs expressing HLA-DR, CD86 and CD40, the expression of CD83 was negligible, suggesting that they are not terminally mature and were likely to retain their macropinocytic activity. Interferon-α, may confer preservation of macropinocytosis, despite enhanced maturity. This is advantageous for immunotherapy, as these DCs are capable of antigen uptake before being inoculated into patients. The allogeneic T cell responses generated by IFN-α containing 4 CCM **-**treated DCs was significantly better than that documented by DCs grown in 2 CCM only. This is probably due to enhanced numbers of DCs expressing HLA class II proteins, CD40 adhesion molecules and co-stimulatory molecule CD86, when prepared with IFN-α.

Secretion of IL-10 was significantly less with the 4 CCM generated DCs, compared with the 2 CCM generated DCs (Figure 5A). In a related study, DCs from cancer patients generated from CD14+monocytes by culturing with GM-CSF and IFN-α, but without IL-4 secreted higher levels of IL-10 (Th2 response), compared with IL-4 and GM-CSF generated DCs [58]. Moreover, the activation/maturation induced by TNF-α, with or without IFN-α, was further underscored by the strongly enhanced production of the pro-inflammatory cytokine IL-12 p40. However, IL-12p70 production was not observed after LPS stimulation in DCs generated with 4 CCM milieu, but significantly enhanced IL-6 production and secretion was seen. This may represent IL-12 exhaustion of DCs which inversely correlates with maturity as shown previously[40,59]. The IL-12p40 subunit exists extracellularly as a monomer (IL12p40) or dimer (IL12(p40)_2_) and can antagonize the action of IL12p70. The concentration of IL12p70 alone is not indicative of IL-12 bioactivity; rather, the bioactivity of IL-12 produced by activated DCs depends on IL12p70, IL12p40, and IL12(p40)_2 _production and their competitive interaction with the IL-12 receptor[60]. This may reflect an underestimation of the IL-12 bioactivity or may be true IL-12 exhaustion of these DCs. This will be clarified as IL-12 bio-activity assessment methods evolve.

Interleukin-12 is an important licensing cytokine that mediates the polarization of activated CD4+ T helper cells to a Th1 phenotype for the generation of potent CD8+ CTL responses. Dendritic cells can be induced *in vitro *and *in vivo *to secrete IL-12, but IL-12 production and secretion is transient and DCs become refractive to subsequent induction of IL-12 release, termed "exhaustion" or "paralysis" [40,61]. These observations strongly suggest that *ex vivo *DC maturation protocols should avoid conditions that induce DCs to produce and secrete IL-12, and should instead use conditions that enable the DCs to acquire responsiveness to IL-12 induction [59].

Despite different (and opposing) biological activities, IFN-α and IL-4 share similar but complementary actions on DC development. The activity of IFN-α documented in our study appears to be distinct from that of previously described effects on DC development. The first preparation of human DCs from haematopoietic progenitors *in vitro *was achieved by growing CD34^+ ^cells in GM-CSF and other cytokines [20]. It was found that the substitution of TNF-α for IFN-α in PBM cultures containing GM-CSF failed to result in generation of cells with a DC immunophenotype. The addition of anti-TNF-α antibodies to cultures containing IFN-α and GM-CSF did not affect the generation of DCs [20]. Ligation of CD40 on CD34^+ ^haematopoietic progenitor cells was shown to induce them to differentiate into DCs [62]. The ability of IL-4 and GM-CSF to differentiate monocytes into DCs *in vitro *is well established [22,63]. Transforming growth factor-β is required for the generation of DCs from CD34^+ ^progenitor cells, but only in serum-free conditions [64]. The addition of either c-*kit *ligand or flt-3 ligand to CD34^+ ^cell cultures containing GM-CSF and TNF-α can augment the yield of DCs but neither affects DC immunophenotype or function [65-67]. Other cytokines have been shown to play a role in DC development *in vitro*. Interleukin-3, like GM-CSF, can cooperate with TNF-α to generate DCs from CD34^+ ^cells [68]. This activity is distinct from that described in this study with IFN-α. Finally, extensive purification of monocytes before culture in IFN-α+GM-CSF had only a minor effect on cell immunophenotype, suggesting that the effect of IFN-α on DC development from monocytes was a direct one [69].

Cutaneous recurrences of primary breast cancers when treated with. injections of natural IFN α and γ, delivered in combination or separately, resulted in complete pathological regression of some of these lesions and was shown to correlate with enhanced expression of HLA-DR in the DCs and consequent increased activation and infiltration of T cells locally. Such an enhanced DC maturation and activation response supports our findings that IFN-α is a pre-requisite for optimal DC maturation and activation in patients with breast cancer [70].

## Conclusion

The ability of IFN-α to induce peripheral blood and lymph node monocyte differentiation into DCs and recovery of tumour-induced DC anergy appears to be distinct from that of previously studied cytokine combinations. We have shown that *ex vivo *DC generation and maturation from monocytes of breast cancer patients is optimally possible in a system of 4 cytokines including IFN-α, which can potentially be used in DC immunotherapy of operable breast cancer patients. *Ex vivo *generated DCs prepared by the system described are currently being administered to patients and immunological responses (both beneficial and regulatory) monitored in an ongoing study which has ethical and MHRA approval [71].

## Methods

### Culture methods

This study was approved by the Lincolnshire local research ethics committee and was carried out according to the guidelines of the World Medical Association, declaration of Helsinki (2004). Written consent was obtained prior to obtaining patient samples. Peripheral blood mononuclear cells were isolated from heparinized blood obtained by venipuncture from 10 patients with operable breast cancer by Histopaque 1.077 (Sigma, UK.) density gradient centrifugation. The blood sample was obtained prior to surgery. The resultant mononuclear cell fraction was washed three times with phosphate-buffered saline (PBS) and re-suspended in complete tissue culture medium (TCM). The latter consisted of RPMI 1640 medium (Sigma, UK.), containing penicillin-streptomycin (100 IU/ml and 100 μg/ml, respectively; Sigma, UK.) and 10% heat-inactivated (56°C for 1 hour) foetal calf serum (FCS) (Sigma, UK.). Similarly, monocytes were isolated from lymph nodes of 10 of these cancer patients. Lymph nodes were injected with 0.9% saline, teased with toothed forceps, mashed and filtered. The remaining fragments were digested for 20(min) minutes at room temperature with 10 ml of collagenase (1 mg/ml; type II, Worthington Biochemical Corp., Freehold, NJ) and 10 ml of DNAase (0.02 mg/ml; grade II bovine pancreatic DNase, Boehringer, Germany) and then treated for 5 min with EDTA to disrupt T cell-DC complexes. Light-density mononuclear were selected by centrifugation. All monocytes were adjusted at 0.5 to 1.0 × 10^7 ^cells/ml, and seeded in 6-well plates for 3 h at 37°C in a 5% CO2 incubator. The non-adherent cells were removed by one gentle wash with pre-warmed (37°C) TCM after 24 hours. The adherent cells were cultured in 2.5 ml of CCM containing recombinant human cytokines-GM-CSF (500 IU/ml, R&D Systems), IL-4 (500 IU/ml, R&D Systems), TNF-α (10 ng/ml, R&D Systems) and IFN-α (500 IU/ml, Sigma) for a further duration of 6 days. The starting population of adherent mononuclear cells was heterogeneous, consisting of CD14^+ ^cells (24 ± 8%), CD3^+ ^cells (63 ± 9%), CD20^+ ^cells (9 ± 4%), and CD56^+ ^cells (12 ± 4%). The effect of GM-CSF+IL-4 (2 CCM), GM-CSF+IL-4+TNF-α (3 CCM), and GM-CSF+IL-4+TNF-α+IFN-α (4 CCM) and medium alone (no cytokine) on the morphological, immunophenotypical and functional characteristics of the adherent culture of monocyte derived-DCs was evaluated. The adherent cell cultures were harvested on day 7 and rinsed gently with TCM. The cultured DCs were enriched by depletion using immuno-magnetic beads (Miltenyi Biotech, Germany) as previously described [15].

### DC enrichment by immunomagnetic depletion

Monocytes were counted in a haemocytometer chamber and made up to a concentration of 1 × 10^7 ^cells/ml with TCM. One ml of monocytes (10^7^cells) was added to a sterile polypropylene FACS tube (Becton Dickinson, UK.), which was centrifuged at 1,200 rpm for 5 min. The supernatant was aspirated and discarded. Thirty μl lineage cocktail containing fluorescein isothiocyanate (FITC)-conjugated MAbs (CD3, CD14, CD16, CD19, CD20 and CD56) from BD BioSciences, UK was then added to the tube. The latter was vortexed and allowed to stand on ice for 30 min in the dark. The tube was again vortexed gently, 1 ml of wash buffer added and the tube centrifuged at 1,200 rpm for 5 min. The supernatant was aspirated and discarded, and the cells gently resuspended in 1 ml of wash buffer [phosphate buffer saline (PBS) containing 0.5% FCS and 0.1 M of EDTA]. The washing procedure was repeated another two times. Following the last wash, the cell pellet was resuspended in 90 μl of PBS. Twenty ml of anti-FITC magnetic microbeads (Miltenyi, Germany) were added to the tube, the contents were mixed well and incubated for 30 min in a refrigerator at 6–12°C. The cells were carefully washed and resuspended in 1 ml of PBS and magnetic separation carried out. A negative depletion column LD was used and placed in the magnetic field (Midi MACs, Miltenyi, Germany). The column was prepared by washing with 3 ml of PBS and a flow resister (25G) was applied at the tip of the column. One ml of the cell suspension was then placed onto the column. The negative cells passed through the column and were collected in the 15 ml conical tube. The column was rinsed 3 times with 2 ml of buffer. The effluent was collected as part of the negative fraction. The enriched DCs were collected by centrifugation at 1,200 rpm for 5 min at RT. The purity of the DCs in the negative fraction was between 75–80%.

### Recombinant human cytokines

The optimal concentration of each cytokine was determined by titration experiments using flow cytometry to quantitate (% cells expressing) CD 40, CD 86 and HLA-DR profiles. Human recombinant IFN-α was obtained from Sigma-Aldrich (London, UK.). This was supplied in vials containing 10 × 10^6 ^IU. The contents of each vial were dissolved in PBS and 0.1% bovine serum albumin (BSA) to yield a stock of 1 × 10^6 ^IU/ml from which subsequent dilutions were made. Human recombinant GM-CSF and IL-4 were obtained from R&D Systems (London, UK.), respectively. Recombinant human TNF-α was obtained from Sigma (London, UK).

### Immunofluorescent staining and flow cytometry

The expression of leukocyte cell surface markers was assessed using the following fluorescein isothiocyanate (FITC)-conjugated murine monoclonal antibodies (mAbs): Lineage cocktail mAbs (CD3, CD14, CD16, CD19, CD20 and CD56) were obtained from Becton Dickinson Systems (Oxford, UK.). The phycoerythrin (PE)-conjugated anti-HLA-DR and CD40, and the APC-conjugated anti-HLA-DR were obtained from Pharmingen, UK. Cy-Chrome™ conjugated anti-CD83 and the ECD conjugated anti-CD86 were obtained from Beckman Coulter, UK. Cell preparations were examined using a 5 multi-colour flow cytometer. Cell suspensions (1 × 10^6 ^cells) were incubated with optimal concentrations of fluorescent-conjugated mAbs for 45 min on ice (0–4°c). The cells were then washed in PBS containing 0.1% BSA and fixed in 3% formaldehyde to exclude dead cells. The EPICS ALTRA flow cytometer (Beckman Coulter, UK.) was used to analyse the cells and establish the phenotypic profiles.

### Evaluation of mannose receptor-mediated endocytosis

Purified DCs (10^5^/sample) were suspended in TCM and placed at either 4° and 37°C. Cascade Blue-conjugated dextran particles (Molecular Probes Inc., UK) were added to each sample at a final concentration of 1 mg/ml and incubation was continued for 45 min. Cells were rinsed twice with cold PBS containing 1% FCS and 0.01% NaN_3_, rinsed once using PBS with 1% paraformaldehyde, and analysed using flow cytometry; violet laser excitation of Cascade Blue and 488 nm excitation of DC phenotyping antibodies occurred as described above. The Cascade Blue-conjugated dextran uptake fluorescence was measured by subtracting the background fluorescence at 4°C from the fluorescence at 37°C. This test of phagocytosis was performed on 10 sets PBDCs derived from monocytes of breast cancer patients cultured under different cytokine conditions.

### Mixed lymphocyte dendritic cell reaction (MLDCR)

Purified T lymphocytes were counted and seeded into plates (96 round-bottomed wells/plate; Costar, Cambridge, UK.) at 10^5 ^cells/well. Purified allogeneic DCs were added to the T cells from healthy donors at 1: 20 in triplicate wells. The final volume of each well was adjusted to 200 μl of TCM. After 5 days of culture, 1 mCi of methyl-[^3^H] thymidine (1 mCi/ml; Sigma, UK) were added to each well and incubation was continued for an additional 18 hours (h). Cells were collected on fibre disks using a Cell Harvester (Packard, UK.) and thymidine uptake was quantitated by liquid scintillation counting [counts per min (cpm)] (Packard Instrumentation, UK.). MLDCR was performed on 10 sets of PBDCs and LNDCs, generated from PBMs and LNMs obtained from 10 patients with operable breast cancer.

### Cytokine analysis of DC cultures and LPS stimulation assay

Supernatants from cultures of adherent PBMs obtained from patients with breast cancer were removed and assayed for cytokine production (IL-10 and IL-12p40), using commercially available quantitative enzyme-linked immunosorbent assay (ELISA) kits (R&D, UK.). The intensity of optical density (OD) was directly proportional to the concentration of the respective cytokines in the sample. The amount of cytokine in each sample was estimated by calibrating the OD values of the standards, supplied with the kits using an ELISA reader. The lowest levels of cytokine detectable by these kits were; IL-10-3.9 pg/ml and IL-12p40 – 5.0 pg/ml.

A further 7 patients with operable breast cancer were recruited and PBDCs were generated with the optimal 4 CCM (GM-CSF+IL-4+TNF-α+IFN-α), which maximally matures DCs. The production and secretion of various cytokines in response to LPS stimulation was studied. The assay was performed in 96 well culture plates containing 50,000 of DCs in 300μl of TCM per well, incubated with 1μg of LPS (LPS, E. Coli, Sigma, UK). Control cultures were set up in the absence of LPS. Culture supernatants were analysed by the cytokine bead array technique (Bender Biomedsystems, Austria) and quantified in pg/ml by comparing the mean fluorescence intensity against a standard curve of diluting standards. The lowest level of cytokine detectable by this kit was 5 pg/ml per cytokine (for IL-12p70, IL-6 and IL-8).

### Statistical methods

SPSS (version 14.0) was used in analysing the data. The unpaired Student-t test was used to analyse the statistical difference between 2 individual samples. When comparisions of more than 2 individual samples were required, analysis of variance (ANOVA) with post hoc Holm-Sidak test was used. A probability value of less than 0.05 (p < 0.05), was considered statistically significant.

## Authors' contributions

SS, MMA, RAR, CV, SC AJM: acquisition of samples and data. MMA, SS, RAR, OE: critically drafting and reviewing the manuscript, including statistical analysis. WV, ME, DV, DC, JAJ: recruitment of patients into the study and reviewing the manuscript. All authors read and approved the final manuscript.
